# Assessing cardiac function in obstructive sleep apnea using a novel metric: integrating the respiratory event frequency and desaturation duration

**DOI:** 10.1186/s12890-025-03974-6

**Published:** 2025-11-12

**Authors:** Ying-Ying Chen, Rachel Chien, Yi-Chih Lin, Wen-Te Liu, Arnab Majumdar, Yen-Ling Chen, Yi-Chun Kuan, Kang-Yun Lee, Po-Hao Feng, Kuan-Yuan Chen, Jiunn-Horng Kang, Hsin-Chien Lee, Cheng-Yu Tsai

**Affiliations:** 1 Research Center of Sleep Medicine, College of Medicine, Taipei Medical University, Taipei, 11031 Taiwan; 2 Center for Artificial Intelligence and Advanced Robotics, National Taiwan University, Taipei, 106319 Taiwan; 3 Department of Otolaryngology, Taipei Medical University-Shuang Ho Hospital, New Taipei City, 23561 Taiwan; 4 School of Respiratory Therapy, College of Medicine, Taipei Medical University, Taipei, 11031 Taiwan; 5 Division of Pulmonary Medicine, Department of Internal Medicine, Taipei Medical University-Shuang Ho Hospital, New Taipei City, 23561 Taiwan; 6 Advanced Technology Lab, Wistron Corporation, Taipei, 11469 Taiwan; 7 Sleep Center, Taipei Medical University-Shuang Ho Hospital, New Taipei City, 23561 Taiwan; 8 Research Center of Artificial Intelligence in Medicine, Taipei Medical University, Taipei, 11031 Taiwan; 9 Department of Civil and Environmental Engineering, Imperial College London, London, SW7 2AZ UK; 10 College of Biomedical Engineering, Taipei Medical University, Taipei, 11031 Taiwan; 11 Department of Neurology, Taipei Medical University-Shuang Ho Hospital, New Taipei City, 23561 Taiwan; 12 Department of Neurology, School of Medicine, College of Medicine, Taipei Medical University, Taipei, 11031 Taiwan; 13 Taipei Neuroscience Institute, Taipei Medical University, Taipei, 11031 Taiwan; 14 Department of Physical Medicine and Rehabilitation, Taipei Medical University Hospital, Taipei, 11031 Taiwan; 15 Graduate Institute of Nanomedicine and Medical Engineering, College of Biomedical Engineering, Taipei Medical University, Taipei, 11031 Taiwan; 16 Department of Psychiatry, Taipei Medical University Hospital, Taipei, 11031 Taiwan; 17 School of Biomedical Engineering, College of Biomedical Engineering, Taipei Medical University, 250 Wuxing Street, Taipei, 11031 Taiwan; 18 Research Center of Thoracic Medicine, Taipei Medical University, Taipei, 11031 Taiwan

**Keywords:** Obstructive sleep apnea, Cardiac function, Left ventricular ejection fraction, Lung-to-finger circulation time, Respiratory event response time area

## Abstract

**Objectives:**

Obstructive sleep apnea (OSA) is associated with impaired cardiac function, evidenced by a decreased left ventricular ejection fraction (LVEF). Traditional measures, including the apnea-hypopnea index (AHI) and lung-to-finger circulation time (LFCT), do not simultaneously consider the hypoxemic duration, frequency, and severity. This study introduces the respiratory event response time area (RERTA), integrating duration- and frequency-based OSA indices, and examined its associations with the LVEF.

**Methods:**

We retrospectively analyzed data from individuals who underwent polysomnography (PSG) and echocardiography within the previous 6 months. LFCT was calculated as the mean time from desaturation onset after a respiratory event to the lowest recorded oxygen saturation (SpO_2_). The RERTA, a joint metric that reflects the event frequency and desaturation duration, was determined as the square root of the product of the AHI and mean LFCT. PSG parameters and related metrics were then examined for associations with echocardiographic measures.

**Results:**

Among 34 participants (10 with mild-to-moderate and 24 with severe OSA), the severe group exhibited a lower median LVEF than the mild-to-moderate group (64.00% [61.00%–68.50%] vs. 70.00% [66.50%–72.75%], *p* < 0.05). After adjusting for age, gender, and the body-mass index (BMI), each 1-event/h increase in the AHI was linked to a borderline reduction in the LVEF (-0.71%, 95% CI: -1.42 to 0.00; *p* = 0.05). A 1-unit increase in the RERTA corresponded to a 0.33% decrease in the LVEF (95% CI: -0.62% to -0.04%, *p* < 0.05).

**Conclusions:**

The RERTA combines the AHI and mean LFCT, providing a joint assessment of circulatory stress in patients with OSA. This study highlights its potential utility in evaluating cardiac function associated with OSA, which can serve as a basis for future prospective research.

**Supplementary Information:**

The online version contains supplementary material available at 10.1186/s12890-025-03974-6.

## Brief summary

To evaluate the impact of obstructive sleep apnea (OSA) on cardiac function, we introduced a composite metric, the respiratory event response time area (RERTA), which integrates the apnea-hypopnea index (AHI) and lung-to-finger circulation time (LFCT). This novel metric jointly considers the respiratory event frequency and desaturation duration, comprehensively assessing the effects of OSA on cardiac function. Data from 34 participants revealed that patients with severe OSA had significantly lower left ventricular ejection fraction (LVEF) values compared to mild-to-moderate cases. Adjusted analyses showed significant associations between increases in the AHI and RERTA with decreases in the LVEF. These findings underscore the potential clinical utility of the RERTA as a indicator for cardiac impairment and circulatory stress in patients with OSA.

## Introduction

Obstructive sleep apnea (OSA) is a prevalent sleep-disordered breathing condition, characterized by repeated episodes of airflow restriction and oxygen desaturation during sleep. A meta-analysis estimated the global prevalence of OSA at 54% [1], with rates of 40%—80% among individuals with cardiovascular disease (CVD) [2]. OSA-induced intermittent hypoxia contributes to myocardial dysfunction and heightened sympathetic activity [3, 4], thereby exacerbating the risk of cardiac deterioration, hospitalization, and mortality [5, 6].

To evaluate the impact of OSA on aggravating cardiac function, previous studies utilized the lung-to-finger circulation time (LFCT), a duration-based index derived from polysomnography (PSG), defined as the time interval from the end of a respiratory event to the lowest recorded oxygen saturation (SpO_2_) [7]. Prior researchers indicated that the LFCT is inversely associated with cardiac output (*p* < 0.01) [8], with an extended LFCT reflecting reduced cardiac output. Additionally, patients with both OSA and heart failure (HF) demonstrated significantly longer LFCT values compared to those without HF (*p* < 0.01) [9]. Those findings suggested that a prolonged LFCT indicated slower circulatory dynamics, a reduced left ventricular ejection fraction (LVEF), and impaired cardiac efficiency [10]. Therefore, the LFCT may serve as a duration-based marker of compromised cardiac function in OSA, offering a more-detailed assessment of cardiovascular stress.

The apnea-hypopnea index (AHI), a frequency-based index commonly used to determine OSA severity, is applied to evaluate the cardiovascular impacts of OSA [11, 12]. Relevant studies indicated that a higher AHI was strongly associated with structural cardiac changes and an increased risk of CVD progression [13, 14]. However, relying solely on frequency-based (e.g., AHI) or duration-based (e.g., LFCT) metrics may overlook the complex interplay among the event frequency, desaturation duration, and hypoxemia severity. Notably, recurrent nocturnal respiratory events accompanied by hypoxemia can activate the sympathetic nervous system, leading to an elevated heart rate and blood pressure, and accelerated blood flow [15]. These compensatory mechanisms may lead to a relative shortening of the LFCT. Therefore, integrating both the respiratory event frequency and oxygen desaturation duration may provide a more-comprehensive evaluation of the OSA-related circulatory burden.

In this study, we proposed a novel composite metric, the respiratory event response time area (RERTA), calculated as the square root of the product of the AHI and mean LFCT (AHI × LFCT)^0.5^. By integrating both the event frequency and desaturation duration, the RERTA may better capture the cardiovascular stress and circulatory burden imposed by OSA. We hypothesized that the RERTA would demonstrate stronger associations with echocardiographic parameters than the AHI or LFCT alone, thereby offering a more-comprehensive indicator of cardiovascular stress in patients with OSA.

## Methods

### Participant enrolment and retrospective collection of data

In this retrospective study, we collected patient lists and relevant medical information in compliance with a protocol approved by the Taipei Medical University (TMU)-Joint Institutional Review Board (IRB; JIRB). Patient data were initially obtained from TMU–Shuang Ho Hospital (New Taipei City, Taiwan). This study first screened the Sleep Center database to identify patients who either had been diagnosed with cardiac function deterioration or were clinically suspected of having cardiac dysfunction. Among these, individuals who had undergone both overnight PSG at the Sleep Center and transthoracic echocardiographic evaluation in the cardiology outpatient clinic following an OSA diagnosis were enrolled. Potential participants were subsequently assessed based on the following inclusion criteria: (1) aged 20–80 years, (2) PSG recording time of >6 h with a sleep efficiency of >60%, (3) no prior invasive surgery for OSA, (4) no use of hypnotic, psychotropic, or sedative medications (e.g., benzodiazepines or opioids), (5) no diagnosis of neuropsychiatric disorders (e.g., neurodegenerative diseases, epilepsy, stroke, schizophrenia, or bipolar disorder), (6) no recent acute cerebrovascular and cardiovascular events, (7) no diagnosis of a chronic illness, such as chronic obstructive pulmonary disease, diabetes, or chronic kidney disease, and (8) PSG and echocardiographic examinations data collected within a 6-month interval. For all eligible patients, additional information—such as age, gender, waist and neck circumferences, height, weight, body-mass index (BMI), smoking status, and comorbidities—was extracted from medical records. The Charlson comorbidity index (CCI) was calculated based on the recorded comorbidities and clinical background data [16].

### PSG parameters and echocardiographic details

Overnight PSG was conducted at a sleep center by certified technologists who continuously monitored patients throughout their stay. Three PSG recording systems, including Embla N7000 (ResMed, San Diego, CA, USA), Embletta MPR (Natus Medical, Pleasanton, CA, USA), and Nox-A1 (Nox Medical, Alpharetta, GA, USA), were employed to collect physiological signals, with scoring using RemLogic software (vers. 3.41; Embla Systems, Thornton, CO, USA). Sleep stages, sleep efficiency, and sleep disorder indices were identified according to American Academy of Sleep Medicine (AASM) guidelines [17]. Specifically, the sleep disorder indices evaluated in this study included the AHI, the total arousal index (ArI), the spontaneous ArI (SpArI), and the respiratory ArI (RArI). Furthermore, to ensure consistent scoring, all PSG data were independently scored by two certified technologists. Inconsistent parts were extracted for further discussion to achieve a consensual decision. Regarding oximetric measures, the study included oxygen desaturation index for ≥ 3% (ODI-3%), the mean SpO_2_, and the proportion of total recording time spent with SpO_2_ below 90% (SpO_2_-<90%).

For echocardiographic parameters, assessments were conducted by cardiologists using the Cardiac Ultrasound System (Philips iE33, Philips, Andover, MA, USA). With the patient in a supine position, the cardiologist positioned the transducer on the anterior thorax and utilized the M-mode to capture sequential cross-sectional images of the ventricle throughout the cardiac cycle. Next, Simpson’s method was applied for 3D reconstruction to derive ventricular volumes from echocardiographic images across multiple planes. A minimum of three consecutive heartbeats were recorded while the patient was at rest, and relevant measurements were averaged. In this retrospective study, the LVEF, left ventricular end-diastolic diameter (LVEDD), and left ventricular end-systolic diameter (LVESD) served as reference indicators for HF [18].

### Definition and calculation of the LFCT and RERTA

**Fig. 1:**
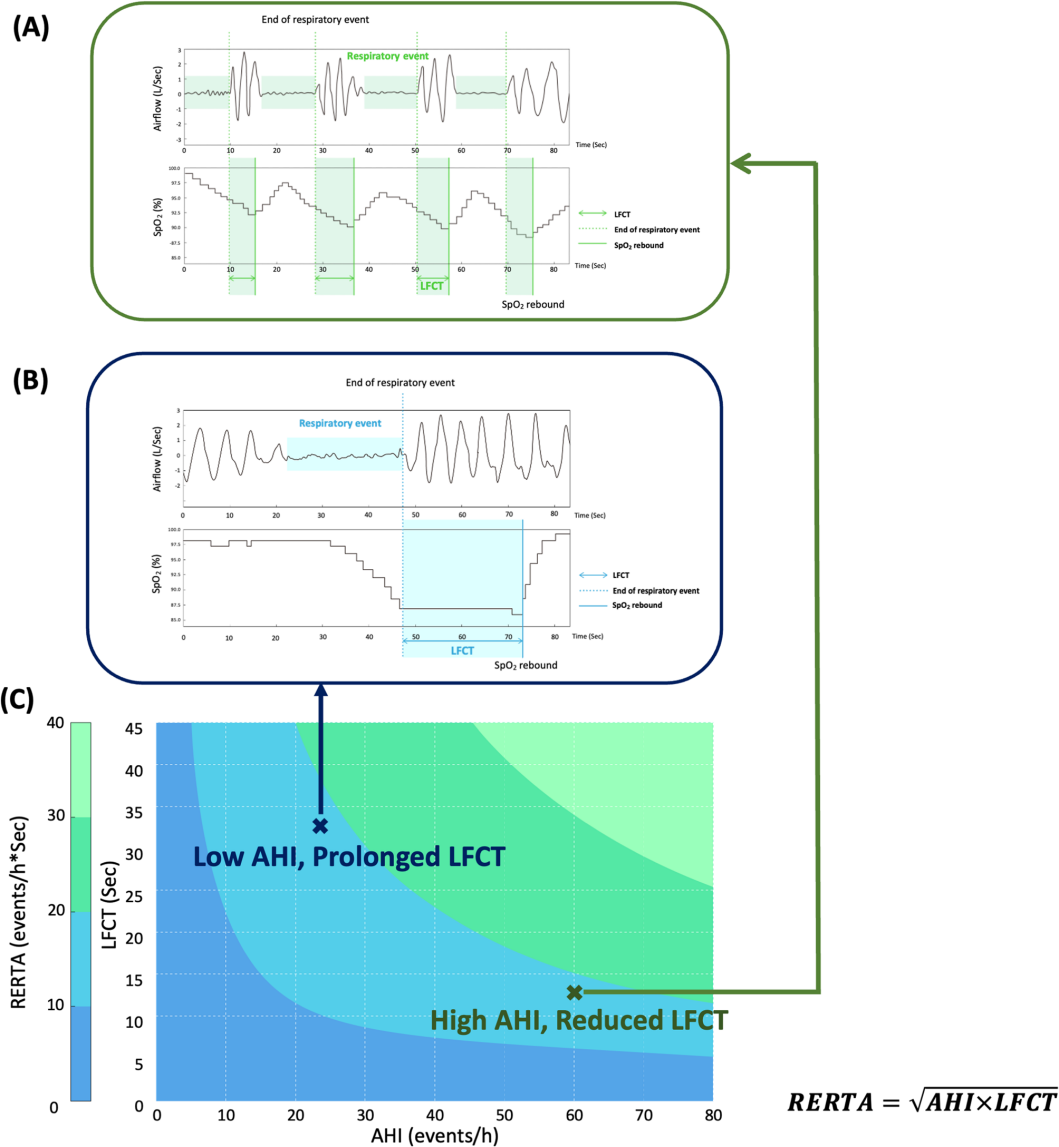
Concept of the respiratory event response time area (RERTA). The concept of the RERTA is an integrated metric that combines the apnea-hypopnea index (AHI) and lung-to-finger circulation time (LFCT) to assess the overall impact of respiratory events and hypoxia in patients with obstructive sleep apnea (OSA). **A** High AHI with short LFCT: frequent respiratory events but rapid recovery from oxygen desaturation. **B** Low AHI with prolonged LFCT: fewer events but extended oxygen desaturation. **C **Contour plot of the RERTA across various AHI and LFCT combinations, showing that a prolonged LFCT with a low AHI can yield a hypoxic burden comparable to, or greater than, a high AHI with a short LFCT—underscoring the importance of considering both the event frequency and duration. Abbreviations: SpO_2_, peripheral oxygen saturation. Note: RERTA is calculated as follows: $\mathrm{RERTA}\;=\sqrt{\mathrm{AHI}\times\mathrm{LFCT}}$

The LFCT, derived from PSG, was defined as the interval beginning at the onset of oxygen desaturation following a respiratory episode (i.e., apnea or hypopnea) and ending at the lowest recorded SpO_2_. Throughout the total sleep period, the LFCT of individuals was determined and averaged to represent the overall LFCT value for subsequent analysis. Although frequent nocturnal oxygen desaturation and an extended LFCT are associated with HF severity [19], frequent respiratory events may paradoxically shorten the LFCT due to sympathetic activation and elevated cardiac output. To capture the bidirectional reciprocal dynamics between the respiratory event frequency and desaturation duration, we introduced a composite metric, the RERTA, calculated as the square root of the product of the LFCT and AHI (Fig. 1**)**. The geometric mean is commonly used in medical research to combine continuous variables while minimizing the influence of extreme values or outliers [20]. Accordingly, the RERTA provides balanced integration of both frequency- and duration-based indices, potentially offering a more-accurate reflection of cumulative circulatory stress among individuals with OSA.

### Statistical analysis

Participants were classified into mild-to-moderate and severe OSA based on an AHI threshold of 30 events/h, in accordance with AASM guidelines [21]. Statistical analyses were performed using Python (vers. 3.6) and the SciPy open-source statistical library (vers. 1.12.0, Python Software Foundation, Fredericksburg, VA, USA). First, the Shapiro-Wilk test was conducted to examine the normality of continuous variables. Normally distributed data were compared using Student’s *t*-test, while the Mann-Whitney U-test was employed for non-normally distributed data. Categorical variables including the smoking status and comorbidities were examined using the Chi-squared test. Next, linear regression analyses were conducted to investigate relationships between clinical measures, using both simple (without adjustment) and multivariable (adjusted for age, sex, and BMI) models. Results are presented as standardized beta coefficients (βs) with 95% confidence intervals (CIs). Statistical significance was set to *p* < 0.05.

## Results

### Baseline characteristics and echocardiographic parameters

This retrospective study included 34 participants with comprehensive clinical data, comprising 10 with mild-to-moderate OSA and 24 with severe OSA. The baseline characteristics, comorbidities, smoking status, and echocardiographic parameters are detailed in Table 1. There were no significant differences between the two groups in terms of age, gender, height, BMI, comorbidities, CCI, or smoking status. Regarding echocardiographic measures, the severe OSA group exhibited significantly lower LVEF levels compared to the mild-to-moderate OSA group (64.00% [61.00%–68.50%] vs. 70.00% [66.50%–72.75%], *p* < 0.05).

**Table 1:** Clinical characteristics and echocardiographic details of participants stratified by the apnea-hypopnea index

Variable	Mild-to-moderate OSA (*n* = 10)	Severe OSA (*n* = 24)	*p* value
Age (years) ^a^	52.50 ± 10.01	47.75 ± 11.78	0.25
Gender (male/female) ^b^	6/4	17/7	0.69
Height (cm) ^a^	166.64 ± 9.09	165.50 ± 7.72	0.73
BMI (kg/m^2^) ^a^	29.46 ± 5.10	32.40 ± 4.72	0.14
Neck circumference (cm) ^c^	39.75 [39.00–42.88]	40.00 [38.00–42.62]	0.91
Waist circumference (cm) ^a^	97.85 ± 13.15	104.47 ± 10.06	0.18
Mean HR (bpm) ^a^	65.30 ± 8.26	70.02 ± 7.99	0.14
Charlson comorbidity index (score) ^c^	2.00 [1.25–2.75]	1.50 [1.00–2.25]	0.79
Comorbidities, ***n*** (%) ^b^			0.97
Hypertension	6 (60.00%)	17 (70.83%)	
Cardiovascular disease	3 (30.00%)	5 (20.83%)	
Metabolic syndrome	4 (40.00%)	9 (37.50%)	
Gastroesophageal reflux disease	2 (20.00%)	6 (25.00%)	
Smoking status,***n*** (%) ^b^			0.19
Current smoker	1 (10.00%)	7 (29.16%)	
Ex-smoker	2 (20.00%)	1 (4.16%)	
Non-smoker	7 (70.00%)	16 (66.67%)	
Echocardiography details			
LVEF (%) ^c^	70.00 [66.50–72.75]	64.00 [61.00–68.50]	0.02
LVEDD (mm) ^a^	49.6 ± 4.35	49.79 ± 9.03	0.93
LVESD (mm) ^c^	30.00 [27.25–32.00]	32.50 [27.00–35.00]	0.27

*OSA* Obstructive sleep apnea, *BMI* Body-mass index, *HR* Heart rate, *LVEF* Left ventricular ejection fraction, *LVEDD* Left ventricular end-diastolic diameter, *LVESD* Left ventricular end-systolic diameter

^a^ Data are expressed as mean ± standard deviation; differences between groups were assessed using Student’s t-test

^b^ Differences between groups were assessed using Fisher’s exact test

^c^ Data are expressed as median [Q1–Q3]; differences between groups were assessed using the Mann–Whitney U-test

### PSG, oximetric measures, and oximetry-related parameters

Table 2. presents the sleep architecture, sleep disorder indices, oximetric measure, and its related parameters of study participants. In terms of sleep architecture, the severe OSA group exhibited a significantly lower proportion of non–rapid eye movement (NREM) sleep relative to total sleep time compared to the mild-to-moderate OSA group (74.70% ± 9.98% vs. 63.88% ± 15.50%, *p* < 0.05). Although there were no significant differences, the severe OSA group exhibited a lower sleep efficiency and reduced total sleep time compared to the mild-to-moderate OSA group. Regarding the sleep disorder index and oximetric measures, the severe OSA group demonstrated significantly higher AHI, oxygen desaturation index (ODI), and RArI values (all *p* < 0.01) compared to the mild-to-moderate OSA group.

**Table 2:** Comparisons of polysomnography, oximetric measures, and oximetry-related parameters among participants stratified by the apnea-hypopnea index (AHI)

Variable	Mild-to-moderate OSA (*n* = 10)	Severe OSA (*n* = 24)	*p* value
Sleep architecture			
Sleep efficiency (%) ^a^	74.84 ± 13.27	66.84 ± 18.92	0.17
Wake (% of SPT) ^b^	12.70 [0.97–18.38]	7.05 [0.28–15.32]	0.89
Total sleep time (min) ^a^	271.88 ± 49.11	250.42 ± 72.80	0.33
REM (% of SPT) ^b^	10.30 [9.15–11.50]	10.15 [6.83–14.40]	0.86
NREM (% of SPT) ^a^	74.70 ± 9.98	63.88 ± 15.50	0.02
Sleep disorder index			
AHI (events/h) ^b^	19.80 [16.45–25.02]	40.05 [33.02–62.47]	< 0.01
ArI (events/h) ^a^	21.23 ± 15.97	28.59 ± 14.38	0.23
SpArI (events/h) ^b^	6.70 [3.27–12.50]	4.35 [3.55–5.75]	0.37
RArI (events/h) ^a^	7.19 ± 5.11	19.27 ± 12.97	< 0.01
Oximetry measures ^b^			
SpO_2_-<90% (%)	2.77 [1.06–15.32]	7.18 [2.03–24.65]	0.23
Mean SpO_2_ (%)	94.25 [92.73–94.97]	93.90 [92.83–95.12]	0.84
ODI-3% (events/h)	16.60 [9.18–18.60]	34.00 [28.05–51.10]	< 0.01
Oximetry-related parameters			
LFCT (s) ^a^	28.23 ± 9.36	21.56 ± 5.00	0.06
RERTA (events/h × s)^0.5 b^	22.43 [18.67–25.61]	29.88 [26.64–32.81]	< 0.01

*OSA* Obstructive sleep apnea, *SPT* sleep period of time, *REM* Rapid eye movement, *NREM* Non–rapid eye movement, *ArI* Arousal index, *SpArI* Spontaneous arousal index, *RArI* Respiratory arousal index, *SpO*_2_-<90% the proportion of total recorded time spent with peripheral arterial oxygen saturation (SpO_2_) < 90%, *ODI-3%* ≥ 3% oxygen desaturation index, *LFCT* the mean of lung to finger circulation during all sleep stages, *RERTA* Respiratory event response time area

^a^ Data are expressed as the mean ± standard deviation; differences between groups were assessed using Student’s *t*-*test*

^b^ Data are expressed as median [Q1-Q3]; differences between groups were assessed using the Mann-Whitney U-test

Additionally, although no significant difference in the LFCT was observed between the two groups, the severe group demonstrated a significantly greater median of RERTA compared to the mild-to-moderate OSA group (22.43 [18.67–25.61] vs. 29.88 [26.64–32.81] (events/h × s)^0.5^, *p* < 0.01).

### Associations of the sleep disorder index with oximetry-related parameters and the LVEF

Table 3. presents associations between the sleep disorder index and LVEF. An increase of 1 event/h in the AHI was associated with a modest decrease of 0.71% in the LVEF (95% CI: −1.42 to 0.00, *p* = 0.05), after adjusting for age, gender, and BMI. Similarly, each 1 event/h increased in the RArI was significantly associated with a 0.61% reduction in the LVEF (95% CI: −1.04 to −0.17, *p* < 0.01). Associations of oximetric measures and related parameters with the LVEF are summarized in Table 4. Among oximetry-related parameters, after adjusting for age, gender, and BMI, a 1-unit increase in the RERTA was significantly associated with a 0.33% decrease in the LVEF (95% CI: −0.62 to −0.04, *p* < 0.05). These associations persisted following adjustment for age, gender, and height (Tables S1, S2).

**Table 3:** Associations between the Sleep Disorder Index and Left Ventricular Ejection Fraction (LVEF)

Variable	LVEF (%)			
	Crude β coefficient (95% CI) ^ac^	*p* value	Adjusted β coefficient(95% CI) ^b^	*p* value
Sleep disorder index				
AHI (events/h)	−0.88 (−1.57 to −0.18)	0.02	−0.71 (−1.42 to 0.00)	0.05
ArI (events/h)	−0.46 (−1.01 to 0.10)	0.10	−0.17 (−0.40 to 0.05)	0.13
SpArI (events/h)	0.20 (−0.09 to 0.49)	0.17	0.19 (−0.12 to 0.50)	0.22
RArI (events/h)	−0.64 (−1.06 to −0.21)	< 0.01	−0.61 (−1.04−0.17)	< 0.01

*CI* Confidence interval, *AHI* Apnea-hypopnea index, *ArI* Arousal index, *SpArI* Spontaneous arousal index, *RArI* Respiratory arousal index

^a^ Simple linear regression models

^b^ Multivariable linear regression models were adjusted for age, gender, and body-mass index

**Table 4:** Associations of oximetric measures and oximetry-related parameters with the Left Ventricular Ejection Fraction (LVEF)

Variable	LVEF (%)			
	Crude β coefficient (95% CI) ^a^	*p* value	Adjusted β coefficient (95% CI) ^b^	*p* value
Oximetry measures				
SpO_2_-<90% (%)	−0.35 (−1.10 to 0.39)	0.34	−0.59 (−1.33 to 0.15)	0.11
Mean SpO_2_ (%)	0.05 (−0.03 to 0.12)	0.23	0.03 (−0.05 to 0.11)	0.43
ODI-3% (events/h)	−0.83 (-1.53 to −0.13)	0.02	−0.63 (−1.33 to 0.08)	0.08
Oximetry-related parameters				
LFCT (s)	−0.05 (−0.33 to 0.22)	0.69	−0.06 (−0.36 to 0.23)	0.66
RERTA (events/h × s)^0.5^	−0.39 (−0.68 to −0.10)	0.01	−0.33 (−0.62 to −0.04)	0.03

*CI* Confidence interval, *SpO*_2_-<90% the proportion of total recorded time spent with peripheral arterial oxygen saturation (SpO_2_) < 90%, *ODI-3%* ≥ 3% oxygen desaturation index, *LFCT* the mean of lung to finger circulation during all sleep stages, *RERTA*, Respiratory event response time area

^a^ Simple linear regression models

^b^ Multivariable linear regression models were adjusted for age, gender, and body-mass index

Table S3. demonstrates the exploratory analysis results comparing optimal cutoff values of the RERTA, AHI, and LFCT across LVEF thresholds of 60% and 70%. The RERTA showed intermediate cutoff values at 29.24 for an LVEF of ≥ 60% and 26.01 for an LVEF of ≥ 70%, relative to those of the AHI and LFCT.

## Discussion

To explore the interplay among OSA severity, hypoxia, and cardiac circulatory function, we examined associations among sleep disorder indices, oximetry-related parameters, and the LVEF across OSA severity levels. Results indicated that despite comparable baseline characteristics and comorbidities, the severe OSA group exhibited significantly lower LVEF, alongside higher AHI and ODI values compared to the mild-to-moderate OSA group. Although not statistically significant, the severe OSA group demonstrated a trend toward lower LFCT. These findings indicated an inverse relationship between the event frequency and desaturation duration. Notably, the novel composite index, RERTA, was significantly elevated in the severe OSA group compared to the mild-to-moderate OSA group. A multivariable linear regression analysis further revealed a significant inverse association between the RERTA and LVEF, independent of age, sex, and BMI.

The severe OSA group demonstrated significantly lower LVEF values compared to the mild-to-moderate group. Several underlying mechanisms may account for this result, including an alteration in the cardiac structure, generation of reactive oxygen species, and activation of the sympathetic nervous system. For instance, previous research demonstrated that OSA-related intermittent hypoxia is linked to endothelial dysfunction and higher oxidative stress [22, 23], predisposing one to compromised cardiac output and reduced circulatory efficiency [24]. Next, intermittent hypoxia activates the sympathetic nervous system, contributing to peripheral vasoconstriction, and an elevated heart rate and blood pressure, thereby deteriorating cardiac health [25]. Several studies indicated that sympathetic overactivity is linked to increased risks of cardiovascular pathologies, such as arterial stiffness and arrhythmias [26, 27]. Overall, the current findings underscore the complex interplay between the respiratory event frequency and physiological compensation in OSA, reinforcing its association with cardiac dysfunction.

The LFCT, which primarily reflects the duration of responding to and accommodating hypoxia, serves as a crucial indicator for evaluating cardiac function in patients with OSA. A trend toward a lower LFCT in the severe OSA group suggests a potential inverse relationship with the AHI. Despite no direct evidence, several reasons may explain this finding. First, as aforementioned, intermittent hypoxia induces excessive sympathetic activation, leading to vasoconstriction, tachycardia, and elevated blood pressure and potentially shortens the LFCT [28]. Second, previous studies demonstrated that a higher hypoxic frequency reduces the baroreflex gain and increases the heart rate, potentially leading to a shorter LFCT [29, 30]. Additionally, a prior study reported that a prolonged LFCT was strongly associated with longer respiratory events, which may in turn reduce the event frequency [31]. While a shortened LFCT reflects an enhanced compensatory response to hypoxia, an increased AHI may contribute to progressive cardiac deterioration. Therefore, these findings underscore the potential of integrating both parameters for cardiovascular risk assessments in patients with OSA.

To comprehensively evaluate the reciprocal dynamics between the LFCT and AHI and their impacts on cardiac function in OSA, the RERTA metric was introduced. Furthermore, to contextualize the clinical feasibility of this novel metric, cutoff values for the RERTA, AHI, and LFCT in classifying the LVEF at the 60% threshold are provided in the Supplementary materials. The severe OSA group exhibited significantly higher mean RERTA values compared to the mild-to-moderate OSA group. Multivariable linear regression models revealed a significant inverse association between the RERTA and LVEF, independent of age, gender, and BMI. Similar results were observed after adjusting for age, gender, and height (Supplementary materials). Given the relatively high mean LVEF (>60%) and small sample size, the modest coefficient may indicate subclinical cardiac impairment. In this cohort, the severe OSA group exhibited a significantly lower LVEF compared to the mild-to-moderate group, suggesting that greater OSA severity may be associated with early declines in cardiac function, even within the normal LVEF range. Previous studies reported an increased risk of stroke and all-cause death among individuals with an LVEF lower than 60%, emphasizing the clinical importance of detecting early functional changes within the normal LVEF range [32, 33]. The geometric mean used to calculate the RERTA captures the cumulative area under the oxygen desaturation curve from all respiratory events, potentially reflecting the hypoxic burden in individuals with OSA [34]. One previous study also demonstrated the utility of geometric mean principles in cardiovascular risk assessments, improving the predictive accuracy by integrating various cardiovascular risk factors [35]. Additionally, an increased hypoxic burden is associated with elevated blood pressure, greater cardiovascular event risks, and all-cause mortality [36, 37]. Collectively, these findings suggest that the RERTA may serve as a clinically relevant indicator for identifying individuals with OSA at increased risk of cardiac dysfunction, highlighting its potential role in risk stratification and guiding clinical decision-making.

The main strength of this study is the introduction of the novel composite RERTA metric, which integrates the AHI (event frequency) and LFCT (desaturation duration) to comprehensively evaluate the impact of OSA on cardiac function. This dual-index approach addresses limitations of relying on a single metric, offering a broader perspective for assessing circulatory stress and cardiovascular risks. The significant inverse association between the RERTA and LVEF further supports its potential as a clinically relevant tool. Additionally, the use of the geometric mean methodology reinforces the utility of mathematically integrating physiological variables into a stable and informative index, in line with prior research. Therefore, these findings provide insights into the assessment of the hypoxic burden and cumulative impacts of the respiratory event frequency and desaturation duration on cardiac function in patients with OSA.

There are several limitations to this study. First, the relatively small sample size (*n* = 34), predominance of participants with severe OSA, and retrospective design may have introduced bias, thereby limiting both the statistical power and generalizability of the current findings. Future prospective studies in larger, independent cohorts are warranted to strengthen the robustness and clinical applicability of the RERTA index. Second, although individuals with acute cerebrovascular and cardiovascular events were excluded, cardiac function assessments solely relied on echocardiography, and the time gap of up to 6 months between the PSG and cardiac evaluations may have led to potential variability in cardiac function. The absence of nocturnal cardiac monitoring may have reduced the precision in capturing hypoxia-related effects. Additionally, given that the mean LVEF exceeded 60% and the majority of participants in both groups had severe OSA, the findings might not be generalizable to populations with reduced LVEF or those with mild to moderate OSA. Future studies should include more-heterogeneous cohorts with a wider range of cardiac phenotypes and OSA severity levels, and should incorporate nocturnal cardiac monitoring, to improve the validity and clinical feasibility of the current results. Third, despite adherence to AASM guidelines, variability in PSG and echocardiography interpretation could have affected the consistency. Future research should adopt automated and standardized scoring systems to improve the data accuracy and consistency. Finally, although the linear regression analyses adjusted for age, sex, and BMI, unmeasured confounders such as lifestyle factors (e.g., smoking, alcohol consumption, and physical activity), use of cardiac medications, socioeconomic status, and baseline cardiopulmonary health may have influenced the results. Future studies could also consider height as an independent confounder. Additionally, non-linear associations between the RERTA and cardiac function were not explored in the present study, which may limit the interpretation of its full clinical relevance. Future research should incorporate non-linear modeling and adjust for additional covariates to enhance the external validity and applicability of the RERTA.

## Conclusions

To comprehensively explore the effects of OSA and hypoxia on cardiac circulatory function, we proposed a novel composite metric of the RERTA, combining the AHI and LFCT. Key findings revealed that patients with severe OSA exhibited significantly lower LVEF and higher AHI and ODI values, compared to those with mild-to-moderate OSA, despite comparable baseline characteristics. A potentially inverse relationship was observed, with the severe OSA group showing a trend toward a lower LFCT. After adjusting for confounders, the RERTA was significantly negatively associated with the LVEF, whereas the AHI demonstrated a borderline inverse association. These findings suggest that the RERTA may serve as an integrated metric of cardiovascular stress in patients with OSA, with potential application in early clinical evaluation of cardiac impairment and risk stratification.

## Supplementary Information

Supplementary Material 1

## Data Availability

Data in this study were collected at the Sleep Canter of Taipei Medical University–Shuang Ho Hospital between July 2023 and July 2024. Because our data include personal information, they are not provided as a supplementary file. Interested parties may contact the corresponding author for access to the dataset and relevant documents.

## Abbreviations

AHI: Apnea-hypopnea index
β: Beta coefficient
BMI: Body-mass index
CI: Confidence interval
CVD: Cardiovascular disease
HF: Heart failure
LFCT: Lung-to-finger circulation time
LVEDD: Left ventricular end-diastolic diameter
LVEF: Left ventricular ejection fraction
LVESD: Left ventricular end-systolic diameter
ODI: Oxygen desaturation index
OSA: Obstructive sleep apnea
PSG: Polysomnography
RERTA: Respiratory event response time area
SD: Standard deviation
SpO_2_: Oxygen saturation
