# Supplementary material for: Assessing cardiac function in obstructive sleep apnea using a novel metric: integrating the respiratory event frequency and desaturation duration
Source: BMC Pulm Med. 2025 Nov 12;25:520. doi: 10.1186/s12890-025-03974-6 (PMC12613519; doi:10.1186/s12890-025-03974-6)
Supplement: Supplementary file 1 — Supplementary Material 1 [file 12890_2025_3974_MOESM1_ESM.docx]

**Supplementary Materials**

To assess the potential influence of height on the left ventricular ejection fraction (LVEF), a multivariate regression analysis was performed with adjustments for age, gender, and height. As shown in **Tables S1** and **S2**, the associations between the sleep disorder index, oximetric measures, and oximetry-related parameters with the LVEF remained consistent with the primary findings of the manuscript. The results suggest that patient height in this cohort might not have exerted a distinct effect compared to the body-mass index (BMI).

**Table S1. Associations between the Sleep Disorder Index and Left Ventricular Ejection Fraction (LVEF)**

| Variable | LVEF (%) | |  |
| --- | --- | --- | --- |
|  | β coefficient (95% CI) ^a^ | *p* value | |
| **Sleep disorder index** |  |  | |
| AHI (events/h) | -0.64 (-1.35 to 0.06) | 0.07 | |
| ArI (events/h) | -0.38 (-0.95 to 0.18) | 0.18 | |
| SpArI (events/h) | 0.21 (-0.11 to 0.52) | 0.19 | |
| RArI (events/h) | -0.56 (-0.99 to -0.14) | 0.01 | |
| Abbreviations: CI, confidence interval; AHI, apnea-hypopnea index; ArI, arousal index; SpArI, spontaneous arousal index; RArI, respiratory arousal index.  The multivariable linear regression models were adjusted for age, gender, and height. | | | |

**Table S2. Associations of Oximetric Measures and Oximetry-related Parameters with the Left Ventricular Ejection Fraction (LVEF)**

| Variable | LVEF (%) | | |
| --- | --- | --- | --- |
|  | β coefficient (95% CI) ^a^ | *p* value |  |
| **Oximetry measures** |  |  |  |
| SpO_2_-<90% (%) | -0.44 (-1.13 to 0.25) | 0.20 |  |
| Mean SpO_2_ (%) | 0.02 (-0.05 to 0.10) | 0.56 |  |
| ODI-3% (events/h) | -0.55 (-1.24 to 0.14) | 0.11 |  |
| **Oximetry-related parameters** |  |  |  |
| LFCT (s) | -0.10 (-0.38 to 0.18) | 0.48 |  |
| RERTA (events/h × s)^0.5^ | -0.32 (-0.62 to -0.02) | 0.04 |  |
| Abbreviations: CI, confidence interval; SpO_2_-<90%, the proportion of the total recorded time spent with peripheral arterial oxygen saturation (SpO_2_) <90%; ODI-3%, ≥3% oxygen desaturation index; LFCT, the mean of lung to finger circulation during all sleep stages; RERTA, respiratory event response time area.  The multivariable linear regression models were adjusted for age, gender, and height. | | |  |

To further evaluate the clinical utility of the respiratory event response time area (RERTA) in individuals with suspected cardiac function deterioration, we conducted exploratory analyses comparing the RERTA with the apnea-hypopnea index (AHI) and

the mean of lung to finger circulation time (LFCT). Specifically, this study utilized Youden's Index to assess the clinical feasibility of the novel RERTA metric, as presented in **Table S3.**

Given the retrospective nature of this study and the requirement that both polysomnography (PSG) and echocardiographic assessments be conducted within a restricted timeframe (<6 months), the number of patients with a left ventricular ejection fraction (LVEF) <50% who could be included was limited. Thus, alternative thresholds of 60% and 70% were applied for the analysis. Notably, prior studies linked an LVEF of <60% with increased risks of stroke and all-cause death. Therefore, identifying metrics that can detect early functional changes within the normal LVEF range is clinically valuable. The RERTA may serve as an early indicator of subclinical cardiac impairment.

Taken together, the RERTA demonstrated intermediate cutoff values at 29.24 (events/h × s)^0.5^ for an LVEF of ≥60% and 26.01 (events/h × s)^0.5^ for an LVEF of ≥70%, compared to the AHI and LFCT.

**Table S3. Comparisons of** **Optimal Cutoff Values Across Different Left Ventricular Ejection Fraction (LVEF) Thresholds**

| Variable | Cutoff value | |  |
| --- | --- | --- | --- |
|  | LVEF ≥60% | LVEF ≥70% |  |
| AHI (events/h) | 40.3 | 33.1 |  |
| LFCT (s) | 22.11 | 21.38 |  |
| RERTA (events/h × s)^0.5^ | 29.24 | 26.01 |  |
| Abbreviations: AHI, apnea-hypopnea index; LFCT, the mean of lung to finger circulation during all sleep stages; RERTA, respiratory event response time area. | | | |
